# Neuroprotective Effects of *Centella asiatica* against Intracerebroventricular Colchicine-Induced Cognitive Impairment and Oxidative Stress

**DOI:** 10.4061/2009/972178

**Published:** 2009-09-13

**Authors:** Anil Kumar, Samrita Dogra, Atish Prakash

**Affiliations:** Pharmacology Division, UGC Center of Advanced Study, University Institute of Pharmaceutical Sciences, Panjab University, Chandigarh 160014, India

## Abstract

Oxidative stress appears to be an early event involved in the pathogenesis of Alzheimer's disease. The present study was designed to investigate the neuroprotective effects of *Centella asiatica* against colchicine-induced memory impairment and oxidative damage in rats. Colchicine (15 *μ*g/5 *μ*L) was administered intracerebroventricularly in the lateral ventricle of male wistar rats. Morris water maze and plus-maze performance tests were used to assess memory performance tasks. Various biochemical parameters such as lipid peroxidation, nitrite, reduced glutathione, glutathione-S-transferase, superoxide dismutase, acetylcholinesterase were also assessed. ICV colchicine resulted marked memory impairment and oxidative damage. Chronic treatment with *Centella asiatica* extract (150 and 300 mg/kg, p.o.) for a period of 25 days, beginning 4 days prior to colchicine administration, significantly attenuated colchicine-induced memory impairment and oxidative damage. Besides, *Centella asiatica* significantly reversed colchicines administered increase in acetylcholinesterase activity. Thus, present study indicates protective effect of *Centella asiatica* against colchicine-induced cognitive impairment and associated oxidative damage.

## 1. Introduction

Alzheimer's disease (AD) is a progressive neurodegenerative disorder and causes significant dementia in elderly. The neuropathological hallmarks of AD include deposits of amyloid *β* fibrils in senile plaques and presence of abnormal tau protein filaments in the form of neurofibrillary tangles [1]. Hippocampus, limbic system, and cortex are the primary areas involved in the pathophysiology of AD [2]. The etiopathogenesis of this disorder is multifactorial and oxidative stress has been reported to play a significant role in the onset and progression of AD. Considering the mechanistic aspects, it has been recognized that *β*-amyloid aggregates and iron accumulation both synergistically cause oxidative damage by free radical generation [3, 4]. Centrally administered colchicine-induced cognitive dysfunction is a well-known model that represents sporadic dementia of Alzheimer's type (SDAT) [5]. Colchicine, a microtubule disrupting agent causes cytoskeletal alterations and axonal transport dysfunction [6] leading to death of cerebellar granule cells, olfactory bulb neurons, cells of subventricular zone, dentate gyrus cells, and basal forebrain cholinergic neurons [7], thus causing cognitive impairment. It induces neurofibrillary degeneration by binding to tubulin, principal structural protein of microtubules [8], thereby inhibiting axoplasmic transport, and mitosis [9]. In addition, central administration of colchicine causes excessive free radical generation and oxidative damage that can be positively correlated with the extent of cognitive impairment [10].

Phytopharmaceuticals are gaining importance as modern medicine as well as traditional system of medicine owing to their therapeutic potential. Novel antioxidants may offer an effective and safe means of bolstering body's defense against free radicals [11] and thereby provide protection against AD like problems. Various natural antioxidants like curcumin, rosmarinic acid, huperzine A have been reported to have a neuroprotective effect against AD [12–14]. *Centella asiatica* (CA) L. Urban (syn. *Hydrocotyle asiatica* L.) belonging to family Apiaceae (Umbelliferae) is a psychoactive medicinal plant being used from centuries in Ayurvedic system of medicine as a *medhya rasayna* [15]. It has been reported to possess various pharmacological effects on CNS such as stimulatory-nervine tonic, rejuvenant, sedative, anxiolytic, and intelligence promoting property [16]. Previous report also demonstrated that *Centella asiatica* leaf extract involved in the morphology of hippocampal CA3 and amygdal neuronal dendritic arborization in neonatal rats. [17, 18]. The whole plant has been shown to improve general mental ability of mentally retarded children [19]. It has also been shown to decrease the oxidative stress parameters [16, 20]. However, its exact mechanism of action in the treatment and management of Alzheimer disease has not been fully understood.

Therefore, present study was designed to investigate the possible neuroprotective effect of *Centella asiatica *against colchicine-induced cognitive impairment and associated oxidative damage in rats.

## 2. Materials and Methods

### 2.1. Animals

Young male Wistar rats (180–200 g) procured from central animal house, Panjab University, Chandigarh were used. Animals were acclimatized to laboratory conditions at room temperature prior to experimentation. Following surgery, animals were kept under standard conditions of a 12-hour light/dark cycle with food and water *ad libitum* in groups of 2, in plastic cages with soft bedding. All the experiments were carried out between 9.00 AM and 3.00 PM. The protocol was approved by the Institutional Animal Ethics Committee of Panjab University, Chandigarh, India, and carried out in accordance with the Indian National Science Academy Guidelines for the use and care of laboratory animals.

#### 2.1.1. Surgery and Intracerebroventricular Administration of Colchicines

Surgery was performed as per the previously described protocol [10]. Animal was anesthetized with thiopental sodium (45 mg/kg) and positioned in a stereotaxic apparatus. The head was positioned in a frame and a midline sagittal incision made in the scalp. Two holes were drilled in the skull for the placement of the injection cannula into both the lateral cerebral ventricles. Co-ordinates for the intracerebroventricular (ICV) cannula implantation were 0.8 mm posterior to bregma, 1.8 mm lateral to the sagittal suture, and 3.6 mm beneath the cortical surface. The scalp was then closed with a suture. Gentamicin (5 mg/kg, IP) was applied to the surgical area in order to prevent sepsis. Animals were housed in a group of two with soft bedding. Special care of the animals was taken during the postoperative period to provide food and water inside the cage of rats. Rats were infused ICV with either artificial cerebrospinal fluid (ACSF; in mmol/l: 147 NaCl, 2.9 KCl, 1.6 MgCl_2, _1.7 CaCl_2_, and 2.2 dextrose) or 15 *μ*g colchicine dissolved in ACSF. Solution (5 *μ*L) was injected using a Hamilton microsyringe positioned in the injection cannula and the syringe was kept in place for 2 minutes in order to allow for the diffusion of the injected volume and prevents pressure-induced damage.

### 2.2. Drugs and Treatment

Colchicine (Sigma chemicals Co., St. Louis, USA) and standardized aqueous extract of *Centella asiatica *(CA) (Dabur Research Foundation, Ghaziabad, India) were used. Colchicine was prepared in ACSF such that a 15 *μ*g dose was delivered in a 5 *μ*L injection volume for ICV administration. For oral administration, aqueous extract of CA was administered in a dose of 0.5 mL/100 g body weight. Animals were divided randomly based on their body weights into seven groups of 7 animals each. The groups were set as shown in Table 1.

The doses of CA aqueous extract were selected based on the previous studies in the laboratory and those reported in the literature.

### 2.3. Behavioral Assessment

#### 2.3.1. Assessment of Cognitive Performance

**Elevated Plus Maze Paradigm**

The elevated plus maze consisted of two opposite black open arms (50 × 10 cm), crossed with two closed walls of the same dimensions with 40 cm high walls. The arms were connected with a central square of dimensions 10 × 10 cm. The entire maze was elevated to a height of 50 cm from the floor. Acquisition of memory was tested on day 13 after colchicine administration. Animal was placed individually at one end of the open arm facing away from the central square. The time taken by the animal to move from the open arm to the closed arm was recorded as the initial transfer latency (ITL). Animal was allowed to explore the maze for 20 seconds after recording the ITL and then returned to the home cage. If the animal did not enter the enclosed arm within 90 seconds, it was guided on the back into one of the enclosed arm and the ITL was given as 90 seconds. Retention of memory was assessed by placing the rat in an open arm and the retention latency was noted on day 14 and day 21 of ITL and was termed as the first retention transfer latency (1st RTL) and second retention transfer latency (2nd RTL), respectively [21].

**Spatial Navigation Task**

The acquisition and retention of a spatial navigation task was evaluated by using Morris water maze [22]. Animals were trained to swim toward a visible platform in a circular pool (180 cm in diameter and 60 cm in height) located in a test room. In principle, rats can escape from swimming by climbing onto the platform and over time the rats apparently learn the spatial location of the platform from any starting position at the circumference of the pool. Thus the platform offers no local cues to guide the escape behavior of the rats. The only spatial cues are those outside of the tank primarily the visual cues. The pool was filled with water (28 ± 2°C) to a height of 40 cm, a movable circular platform (9 cm diameter), mounted on a column, was placed in a pool 2 cm above the water level during the acquisition phase. A similar platform was placed in the pool 2 cm below the water level for the maze retention phase. The water was made opaque by adding a nontoxic dye. Four equally spaced locations around the edge of the pool (N, S, E, and W) were used as starting points and this divided the pool into four equal quadrants.

(1) *Maze acquisition phase (training)*. Animals received a training session consisting of 4 trials on day 13. In all 4 trials, the starting position was different. A trial began by releasing the animal into the maze facing towards the wall of the pool. The latency to find the escape platform was recorded to a maximum of 90 seconds. If the rat did not escape onto the platform within this time, it was guided to the platform and was allowed to remain there for 20 seconds. The time taken by rat to reach the platform was taken as the initial acquisition latency (IAL).

(2) *Maze retention phase (testing for retention of the learned task).* Following 24 hour (day 14) and 8 days (day 21) after IAL, the rat was released randomly from one of the edges facing the wall of the pool. The time taken to find the hidden platform was recorded and termed as first retention latency (1st RL) and second retention latency (2nd RL) on day 14 and day 21 following central administration of colchicines, respectively.

#### 2.3.2. Assessment of Gross Behavioral Activity

Gross behavioral activity was observed on days 1, 7, 14, and 21 following ICV colchicine injection. Animal was placed in a square (30 cm) closed arena equipped with infrared light-sensitive photocells using digital photoactometer. The animals were observed for a period of 5 minutes and the values were expressed as counts/5 minutes [23].

### 2.4. Dissection and Homogenization

On day 24, after behavioral assessments, animals were scarified by decapitation prior to deep anesthesia. The brains were removed, forebrain was dissected out, and cerebellum was discarded. Brain was put on ice and rinsed with ice-cold isotonic saline. A (10% w/v) homogenate was prepared in 0.1 M phosphate buffer (*p*H 7.4). The homogenate was centrifuged at 10,000  g for 15 minutes and aliquots of supernatant was separated and used for biochemical estimation.

### 2.5. Biochemical Tests

#### 2.5.1. Measurement of Lipid Peroxidation

The extent of lipid peroxidation in the brain was determined as described by Wills [24]. The amount of malondialdehyde (MDA) was measured by reaction with thiobarbituric acid at 532 nm using Perkin Elmer Lambda 20 spectrophotometer. The values were calculated using the molar extinction coefficient of chromophore (1.56 × 10^5 ^(mol/L)^−1^cm^−1^).

#### 2.5.2. Estimation of Reduced Glutathione

Reduced glutathione was estimated according to Ellman [25]. A 1-mL supernatant was precipitated with 1 mL of 4% sulphosalicylic acid and cold digested for 1 hour at 4°C. The samples were then centrifuged at 1,200 g for 15 minutes at 4°C. To 1 mL of the supernatant obtained, 2.7 mL of phosphate buffer (0.1 mmol/L, *p*H 8) and 0.2 mL of 5, 5′ dithio-bis (2-nitrobenzoic acid) (DTNB) was added. The developed yellow color was measured at 412 nm using Perkin Elmer Lambda 20 spectrophotometer. Results were calculated using the molar extinction co-efficient of the chromophore (1.36 × 10^4^ (mol/L)^−1^cm^−1^).

#### 2.5.3. Estimation of Nitrite

The accumulation of nitrite in the supernatant, an indicator of the production of nitric oxide, was determined by a colorimetric assay with Greiss reagent according to Green et al. [26]. The absorbance was measured at 540 nm using Perkin Elmer Lambda 20 spectrophotometer. The concentration of nitrite in the supernatant was determined from sodium nitrite standard curve.

#### 2.5.4. Superoxide Dismutase Activity

Superoxide dismutase (SOD) activity was assayed by the method of Kono [27]. The assay system consisted of EDTA 0.1 mM, sodium carbonate 50 mM and 96 mM of nitro blue tetrazolium (NBT). In the cuvette, 2 mL of the above mixture, 0.05 mL of hydroxylamine, and 0.05 mL of the supernatant were added, and the auto-oxidation of hydroxylamine was measured for 2 minutes at 30-second interval by measuring the absorbance at 560 nm using Perkin Elmer Lambda 20 spectrophotometer.

#### 2.5.5. Catalase Activity

Catalase activity was assessed by the method of Luck [28], wherein the breakdown of hydrogen peroxide is measured. Briefly, the assay mixture consisted of 3 mL of H_2_O_2 _ phosphate buffer and 0.05 mL of the supernatant of the tissue homogenate. The change in absorbance was recorded for 2 minutes at 30-second interval at 240 nm using Perkin Elmer Lambda 20 spectrophotometer. The results were expressed as micromoles of H_2_O_2_ decomposed per minute per mg protein.

#### 2.5.6. Glutathione-S-Transferase Activity

The activity of glutathione-S-transferase was assayed by the method of Habig and Jakoby [29]. Briefly, the assay mixture consisted of 2.7 mL of phosphate buffer, 0.1 mL of reduced glutathione, 0.1 mL of 1-chloro-2, 4-dinitrobenzene (CDNB) as substrate, and 0.1 mL of supernatant. The increase in the absorbance was recorded at 340 nm for 5 minutes at 1-minute interval using Perkin Elmer Lambda 20 spectrophotometer. The results were expressed as nmoles of CDNB conjugated/min/mg protein.

#### 2.5.7. Acetyl Cholinesterase (AChE) Activity

Acetyl cholinesterase (AChE) is a marker of extensive loss of cholinergic neurons in the forebrain. The AChE activity was assessed by Ellman method [30]. The change in absorbance was measured for 2 minutes at 30-second interval at 412 nm using Perkin Elmer Lambda 20 spectrophotometer. Results were expressed as micromoles of acetylthiocholine iodide hydrolyzed per minute per mg protein.

#### 2.5.8. Protein Estimation

The protein content was estimated by biuret method [31] using bovine serum albumin as a standard.

### 2.6. Statistical Analysis

Values are expressed as mean ± SEM. The behavioral assessment data were analyzed by a repeated measures two-way ANOVA with drug-treated groups as between and sessions as the within-subjects factors. The biochemical estimations were separately analyzed by one-way ANOVA. Post-hoc comparisons between groups were made using Tukey's test. The value *P* < .05 was considered significant.

## 3. Results

### 3.1. Centella asiatica (CA) Improved on Behavioral Alteration in Colchicine Treated Rats

#### 3.1.1. Elevated Plus Maze

In the present experiment, mean ITL on day 13 for each rat was relatively stable and showed no significant variation among different groups. All the rats entered the closed arm within 90 seconds. Following training, sham-operated, ACSF-injected, and CA-treated (150 and 300 mg/kg, PO) rats entered closed arm quickly as compared to colchicine treated rats. Mean retention transfer latencies (1st RTL and 2nd RTL) to enter closed arm on days 14 and 21 were shorter as compared to ITL on day 13 of each group, respectively. In contrast, colchicine-injected rats performed poorly throughout the experiment and did not show any change in the mean retention transfer latencies on days 14 and 21 as compared to pretraining latency on day 13, demonstrating that colchicines-induced marked memory impairment. Chronic administration of CA (150 and 300 mg/kg) beginning prior to colchicine injection significantly decreased the mean retention latencies on days 14 and 21 following colchicine injection (*P* < .05 versus ICV colchicine group) (Table 2). The mean transfer latencies of CA- treated (150 and 300 mg/kg, PO) and ICV colchicines-treated groups were significantly different from that of CA per se groups on days 14 and 21 (*P* < .05) (Table 2).

#### 3.1.2. Morris Water Maze

Sham-operated, ACSF-injected, and CA per se (150 and 300 mg/kg, PO) group of animals quickly learned to swim directly to the platform in the Morris water maze on day 13. Colchicine-treated rats showed an initial increase in escape latency, which declined with continued training during the acquisition of a spatial navigation task on day 13. CA (150 and 300 mg/kg, PO) group of rats was also performed similarly during the acquisition of a spatial navigation task on day 13 (versus ACSF-injected group). There was a significant difference in the mean IAL of colchicines-treated group compared to ACSF-injected group on day 13 indicating colchicine-induced impaired acquisition of spatial navigation task (*P* < .05). In contrast, CA (150 and 300 mg/kg, PO) treatment significantly decreased the IAL to reach the platform in the pretrained rats as compared to colchicine treated rats on day 13 following colchicine administration (Table 3).

Following training, the mean retention latencies (1st and 2nd RL) to escape onto the hidden platform were significantly decreased in sham-operated and ACSF-injected rats on days 14 and 21, respectively, as compared to IAL on day 13 following colchicine administration. On the contrary, the performance in the colchicines-treated rats was changed after initial training in the water maze on days 14 and 21, with significant increase in mean retention latencies compared to IAL on day 13. The results suggest that colchicine caused significant cognitive impairment. However, chronic CA treatment (150 and 300 mg/kg, PO) starting before colchicine administration showed a significant decline in the 1st and 2nd RL as compared to colchicines-treated rats on days 14 and 21, respectively, following colchicine administration (Table 3) and improved the retention performance of the spatial navigation task.

### 3.2. Effect of Centella Asiatica on Locomotor Activity

**Figure 1:**
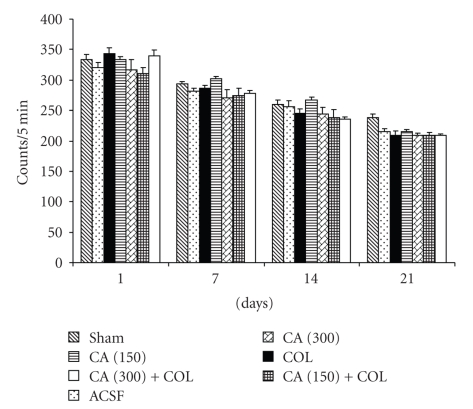
*Centella asiatica* (CA; 150 and 300 mg/kg, PO) on locomotor activity in intracerebroventricular colchicines- (COL-) injected rats. Values are mean ± SEM. Data was analyzed by two-way anova (*n* = 12 in each group).

In the present series of experiments, the mean scores of locomotor activity for each rat were relatively stable and showed no significant variation among different groups. The mean scores in sham-operated, ACSF-, and colchicines-treated rats remained unchanged. Further, both the dose of CA (150 and 300 mg/kg, PO) did not cause any significant alteration in the locomotor activity as compared to colchicine treated rats on days 14 and 21 (Figure 1).

### 3.3. Antioxidant Effect of Centella Asiatica (CA) in Colchicine-Treated Rats

Central administration of colchicine caused significant rise in brain MDA, nitrite levels, depletion of GSH, glutathione-S-transferase, SOD, and catalase levels as compared to ACSF. However, chronic CA (150 and 300 mg/kg, PO) treatment significantly attenuated the increase in MDA, nitrite levels, and restored decrease in reduced GSH (Table 3). CA treatment also caused a significant increase in glutathione-S-transferase, SOD, and catalase levels (Table 4).

### 3.4. Reversal of Increased Brain Acetylcholine Levels by Centella Asiatica in Colchicine-Treated Rats

**Figure 2:**
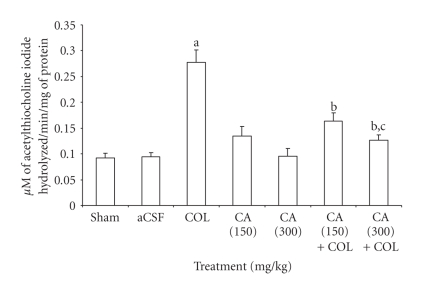
*Centella asiatica* (CA; 150 and 300 mg/kg, PO) on acetyl cholinesterase activity in Intracerebroventricular colchicines- (COL-) treated rats. Values are mean ± SEM; ^a^ *P* < .05 as compared to artificial cerebrospinal fluid- (aCSF-) injected group; ^b^ *P* < .05 as compared to colchicine treated group; ^c^ *P* < .05 as compared to CA (150) + COL group; (one-way ANOVA followed by Turkey's test for multiple comparisons). Note: aCSF: artificial cerebrospinal fluid; COL: colchicine; CA: *Centella asiatica *

Intracerebroventricular administration of ACSF did not show any significant effect on brain acetylcholinesterase levels as compared to sham-operated rats. In contrast, central colchicine injection showed significant increase in the brain AChE activity as compared to ACSF-injected rats. However, chronic oral administration of CA (150 mg/kg and 300 mg/kg, PO) significantly attenuated enhanced AChE activity compared to colchicines-treated group (Figure 2).

## 4. Discussion

The present study investigated the effect of *Centella asiatica* (CA) extract in the prevention of sporadic dementia of Alzheimer's type using intracerebroventricular colchicines-induced rats. Salient findings of this study are that pre- and postcolchicine treatment with CA improved cognition, decreased malondialdehyde, and nitrite levels, restored decrease in GSH, increased activities of glutathione-S-transferase, catalase, and SOD. This illustrates that central administration of colchicine is characterized by progressive deterioration of learning and memory, oxidative stress, and decrease in acetylcholine turnover [32, 33]. Cytoskeletal disruption has been linked to neurodegeneration in AD [34]. Colchicine is an alkaloid derivative that binds irreversibly to microtubules and causes their depolymerization, thereby inhibiting their assembly. This leads to impaired intracellular trafficking of neurotrophic factors, synaptic loss, and increased axonal excitotoxicity [35].

In the present study, colchicine when given centrally resulted in significant memory impairment in elevated plus maze and Morris water maze tasks which were attenuated by chronic CA treatment. In the present study, chronic administration of CA was able to improve the cognitive deficit and attenuated oxidative stress, suggesting that CA improves cognitive task and has antioxidant-like effect [16, 36]. Additionally, CA leaf extract has been reported to improve spatial learning performance and enhance memory retention in neonatal rats during growth spurt period and also found efficient in enhancing hippocampal CA_3_ neuronaldendritic arborization in rats [18, 37].

Lipid peroxidation plays a major role in oxidative damage of lipids. The key metabolites of lipid oxidation are malondialdehyde (MDA) and 4-hydroxynonenal (HNE). It has been reported that the level of MDA are generally higher in AD. Further, it has been supported that the level of HNE also found abundantly in on apolipoprotein E in vitro and on cyto skeletal proteins in cell culture [38]. Our results also proved that the administration of colchicine produced the increased MDA levels which are more responsible for the oxidative damage in rats.

A growing body of evidence supports the fact that free radicals are the most likely candidates responsible for producing neuronal changes mediating the behavioral deficits in AD [39, 40]. In fact, there exists a close correlation between oxidative stress and A*β* deposition [41]. Although colchicine is one of the major oxidative medication of proteins resulting from peroxynitrite which is associated with free radical and nitric oxide, central administration of colchicine causes oxidative stress by increasing GLU/GABA ratio [42] and increasing NOS production in the brain [43] . This results in an excessive glutamate activity [44] and NO production thereby resulting in oxidative stress and extensive neuronal damage [45]. NOS-containing neurons are relatively wide spread in AD. It has been reported that activated microglia are widely abundant in most senile plaque in AD which are responsible for the production of nitric oxide [46] . So it seems that the production of nitric oxide produced by oxidative stress with colchicine is an additional link to lower incidence of AD with use of antioxidant agent. 

In the present study, CA per se did not show any significant effect on the oxidative stress markers in the brain of normal animals. However, CA treatment significantly attenuated the colchicines-induced oxidative stress. The main chemical constituents of CA are triterpenes mainly pentacyclic triterpenic acids and their respective glycosides, including asiatic acid, asiaticoside, madecassic acid, madecassoside, brahmoside, brahmic acid, brahminoside, thankuniside, isothankuniside, centelloside, madasiatic acid, centic acid, and cenellicacid [47]. Beside triterpenoids and essential oils, CA has also been reported to contain numerous flavonoids, including quercetin, kaempferol, catechin, rutin, and naringin, some of which are major contributors in particular to the antioxidative activity of CA [48]. CA has been recently indicated to show antilipid peroxidative and free radical scavenging activities [49, 50].

Glutathione is an endogenous antioxidant presenting in the reduced form within the cells. It has been shown to react with free radicals and prevent generation of hydroxyl free radicals [51]. The decreased level of GSH and glutathione-S-transferase activity in colchicines-treated animals indicates that there is an increased generation of free radicals and reduced activity of glutathione system in combating oxidative stress. CA treatment was able to restore the GSH levels and also cause a significant increase in the glutathione-S-transferase activity. Central administration of colchicine causes an increase in expression of NOS resulting in increased levels of NO which is neurotoxic to cholinergic neurons [52]. Nitric oxide also acts as a precursor for peroxynitrite free radical which results in neuronal damage. This explains that central administration of colchicine caused a significant increase in the nitrite levels in the brain and CA treatment was able to decrease the raised nitrite levels. Colchicine also induces a direct inflammatory response in the CNS [53] which causes cholinotoxicity. Jin et al. [54] showed that CA aqueous extract which contains asiaticoside has an anti-inflammatory property that is brought about by inhibition of NO synthesis. This may also have a role in explaining the neuroprotective effect of CA.

Central administration of colchicine produces marked destruction of hippocampal granule cells and septohippocampal pathways resulting in loss of cholinergic neurons and decreased activities of acetylcholinesterase and choline acetyltransferase [8]. In the present study, colchicine caused a significant increase in the acetylcholinesterase activity thereby leading to learning and memory deficits. CA was able to ameliorate the colchicine induced decrease in AChE activity. In summary, the present study suggests that chronic administration of CA prevents colchicine-induced cognitive impairment and associated oxidative stress. Thus, the use of CA is promising for the treatment of AD and other neurodegenerative disorders.

## Figures and Tables

**Table 1:** 

Group number	Treatment
(1)	Sham-operated (vehicle for CA)
(2)	ACSF (5 *μ*L, ICV ) + vehicle for CA
(3)	Colchicine (15 *μ*g/5 *μ*L) + vehicle for CA
(4)	CA (150 mg/kg, PO) + ACSF
(5)	CA (300 mg/kg, PO) + ACSF
(6)	CA (150 mg/kg, PO) + colchicine
(7)	CA (300 mg/kg, PO) + colchicine

**Table 2:** Effect of *Centella asiatica* (CA; 150 and 300 mg/kg, PO) on memory performance in elevated plus maze paradigm in intracerebroventricular colchicines- (COL-) injected rats.

Treatment (mg/kg)	Mean transfer latency (in seconds)		
	ITL	1st RTL	2nd RTL
Sham	58.16 ± 1.79	20.5 ± 4.28	17.0 ± 5.16
ACSF	61.16 ± 1.6	15.66 ± 1.66	12.60 ± 1.49
COL	66.66 ± 1.53^a^	79.33 ± 1.33^a^	72.33 ± 1.20^a^
CA (150)	62.16 ± 1.51	19.16 ± 0.945	13.16 ± 1.16
CA (300)	61.8 ± 1.077	17.16 ± 1.79	10.16 ± 0.844
CA (150) + COL	66.3 ± 1.470	49.33 ± 0.881^b^	42.8 ± 0.478^b^
CA (300) + COL	62.8 ± 1.238^c^	33.6 ± 0.577^b,c^	29.44 ± 0.958^b,c^

The initial transfer latencies (ITLs) on day 13 and retention transfer latencies on days 14 (1st RTL) and 21 (2nd RTL) following colchicine injection were observed. Values are mean ± SEM; ^a^ *P* < .05 as compared to artificial cerebrospinal fluid (ACSF)-injected group; ^b^ *P* < .05 as compared to colchicine-injected group; ^c^ *P* < .05 as compared to CA (150) + COL group; (repeated measures two-way ANOVA followed by Tukey's test for multiple comparisons).

Note: ACSF: artificial cerebrospinal fluid; COL: colchicine; CA: *Centella. asiatica*

**Table 3:** Effect of *Centella asiatica* (CA; 150 and 300 mg/kg, PO) on spatial navigation task in intracerebroventricular colchicines- (COL-) injected rats.

Treatment (mg/kg)	Mean latency (in seconds)		
	IAL	1st RL	2nd RL
Sham	43.5 ± 1.29	12.33 ± 1.4	9.16 ± 2.16
ACSF	55.33 ± 1.7	14.33 ± 1.66	11.8 ± 1.49
COL	88.0 ± 1.93^a^	75.0 ± 1.43^a^	62.33 ± 1.80^a^
CA (150)	62.33 ± 1.51	12.83 ± 1.945	10.16 ± 2.16
CA (300)	60.8 ± 1.077	11.83 ± 1.09	9.66 ± 1.421
CA (150) + COL	70.6 ± 2.470^b^	40.0 ± 1.577^b^	32.15 ± 0.475^b^
CA (300) + COL	66.66 ± 1.438^b,c^	26.0 ± 0.569^b,c^	21.5 ± 1.576^b,c^

The initial acquisition latencies (IALs) on day 13 and retention latencies on days 14 (1st RL) and 21 (2nd RL) following colchicine injection were observed in Morris water maze. Values are mean ± SEM; ^a^ *P* < .05 as compared to artificial cerebrospinal fluid- (ACSF) injected group; ^b^ *P* < .05 as compared to colchicine-injected group; ^c^ *P* < .05 as compared to CA (150) + COL group; (Repeated measures two-way ANOVA followed by Tukey's test for multiple comparisons).

Note: ACSF: artificial cerebrospinal fluid; COL: colchicine; CA: *Centella asiatica.*

**Table 4:** Effect of *Centella asiatica* (CA; 150 and 300 mg/kg, PO) on colchicines-induced oxidative stress parameters in rat brain.

Treatment (mg/kg)	MDA levels nmol MDA/mg protein (% of sham)	Nitrite levels *μ*mol/mg protein (% of sham)	Reduced glutathione nmol/mg protein (% of sham)	Catalase *μ*mol of hydrogen peroxide decomposed/min/mg protein ( % of sham)	Superoxide dismutase Units/mg protein (% of sham)	Glutathione- S- transferase nmol of CDNB conjugated/min/mg protein (% of sham)
Sham	100 ± 10	100 ± 12	100 ± 10	100 ± 12	100 ± 14	100 ± 12
ACSF	114.28 ± 11	109.33 ± 11	103.33 ± 11	97.05 ± 10	97.52 ± 15	98.66 ± 16
COL	339.88 ± 32^a^	298.39 ± 15^a^	23.38 ± 10^a^	17.98 ± 8^a^	13.47 ± 3^a^	18.67 ± 7^a^
CA (150)	106.7 ± 15	101.29 ± 16	90.35 ± 17	93.9 ± 13.7	99.17 ± 17	97.64 ± 14
CA (300)	123.4 ± 28	99.44 ± 26	93.77 ± 15	98.8 ± 10.8	98.16 ± 16	98.65 ± 13
CA (150) + COL	229.81 ± 27^b^	210.91 ± 8^b^	50.45 ± 8^b^	36.96 ± 6^b^	39.05 ± 10^b	30.65 ± 8^b^
CA (300) + COL	157.26 ± 16^b,c^	148.98 ± 7.9^b,c^	81.61 ± 7.6^b,c^	69.71 ± 5^b,c^	65.52 ± 5^b,c^	65.05 ± 6^b,c^

Values are mean ± SEM; ^a^ *P* < .05 as compared to artificial cerebrospinal fluid- (ACSF-) injected group; ^b^ *P* < .05 as compared to colchicine-injected group; ^c^ *P* < .05 as compared to CA (150) + COL group; (repeated measures two-way ANOVA followed by Tukey's test for multiple comparisons).

Note: ACSF: artificial cerebrospinal fluid; COL: colchicine; CA: *Centella asiatica.*
